# Personality Factors and Sick Leave Days. Evidence from a Nationally Representative Longitudinal Study in Germany.

**DOI:** 10.3390/ijerph17031089

**Published:** 2020-02-09

**Authors:** Yulia I. Raynik, Hans-Helmut König, André Hajek

**Affiliations:** Department of Health Economics and Health Services Research, University Medical Center Hamburg-Eppendorf, 20246 Hamburg, Germany; h.koenig@uke.de (H.-H.K.); a.hajek@uke.de (A.H.)

**Keywords:** personality, sick leave days, GSOEP, longitudinal studies, public mental health (PMH)

## Abstract

Background: The question of whether employees’ sickness absence from the workplace depends on personality has been researched. Existing evidence mostly stems from cross-sectional studies, mainly showing that personality factors were not associated with the number of sick leave days, except for neuroticism, which was positively associated with sick leave days. Based on the above, it remains an under researched question whether intraindividual changes in personality factors are associated with changes in sick leave days. Thus, based on a nationally representative sample, the current study aimed to investigate the relationship between personality factors and sick leave days longitudinally based on a nationally representative sample of individuals in Germany. Methods: The present study used data from the German Socio-Economic Panel (GSOEP), a longitudinal survey of private households in Germany. Information from the years 2005, 2009, 2013 were used. The Big Five Inventory-GSOEP (BFI-S) was used to measure personality. Sick leave days in the preceding year were recorded. Poisson fixed effects regressions were used. Results: Adjusting for potential confounders, regressions showed that increases in neuroticism were associated with increased sick leave days. The longitudinal association between extraversion and sick leave days was marginally significant (*p* < 0.10). Other personality factors were not significantly associated with sick leave days. In addition, sick leave days increased with worsening self-rated health, presence of severe disability and increasing age. Conclusions: The findings of the current study highlight the association between neuroticism and sick leave days longitudinally. Further research is required to elucidate the underlying mechanisms.

## 1. Introduction

In recent decades, interest in research on personality has been increasing, because, among others, research on the topic of personality repeatedly shows the importance of this aspect in various economic aspects [1,2,3,4,5]. Thereby, the so-called “Big Five” approach is widely used to describe personality factors. According to it, personality differences between individuals can be divided into five basic personality traits: neuroticism, extraversion, openness to experience, agreeableness and conscientiousness. Judge and his colleagues [6] describe these five factors as follows: neuroticism signifies perception of, in particular, negative emotions and is associated with anxiety and emotional instability, extraversion signifies perceptions and behaviors in relationships between people, extraverted people are often characterized as sociable, enthusiastic and energetic, openness to experience signifies intelligence, open-mindedness and a tendency to be non-conforming, agreeableness is associated with the ability to build teams, to care about others, and conscientiousness refers to job performance, to what extent is the person organized, unimpulsive and reliable. In addition, personality changes at least to some degree throughout the life of an individual [7]. 

The question of whether employees’ sickness absence from the workplace depends on personality has also been researched [8,9,10]. Existing evidence mostly stems from cross-sectional studies, mainly showing that personality factors were not associated with the number of sick leave days, except for neuroticism, which was positively associated with sick leave days. The only exception is the longitudinal study of Kok and colleagues [11]. Using multiple logistic regressions, they investigated whether conscientiousness influences long-term absenteeism (11 and more working days). Based on the above, it remains an under researched question whether intraindividual changes in personality factors are associated with changes in sick leave days. Thus, based on a nationally representative sample (Germany), the current study aimed to investigate the relationship between personality factors and sick leave days longitudinally.

## 2. Materials and Methods 

### 2.1. Sample

For this study, data were derived from three survey waves (22th wave taking place in the year 2005, 26th wave taking place in the year 2009, and 30th wave taking place in the year 2013) of the German Socio-Economic Panel (GSOEP). The GSOEP is the largest household panel study in Germany. Since 1984 (first survey wave), every year Germans (from Old and New German States), foreigners, and immigrants have been interviewed annually. The data covers practically all spheres of the participants’ lives: education, employment, income, health, satisfaction indicators and others. Further details are given elsewhere [12]. In this study, we restricted our analysis to employed individuals aged 17 to 64 years. 

### 2.2. Dependent Variables

The number of sick leave days in the preceding year was reported by the respondents. 

### 2.3. Independent Variables

Based on the Big Five Inventory (BFI), Rammstedt et al. [13] developed a short scale to quantify the personality factors (Big Five Inventory-SOEP, BFI-S), which consists of 15 items, especially for the German-speaking area. It has been demonstrated that this scale has satisfactory psychometric properties. [14] According to the BFI-S, each personality factor is measured using three items (by summing the three items); each item represents one statement, which can be evaluated with a seven-point answer scale (1= “not correct at all” to 7= “fully correct”). Each subscale ranges from 3 to 21. With regard to the neuroticism, higher values indicate higher neuroticism. With regard to extraversion, higher values indicate higher extraversion. With regard to openness to experience, higher values indicate higher openness to experience. With regard to agreeableness, higher values indicate higher agreeableness. With regard to conscientiousness, higher values indicate higher conscientiousness. 

Moreover, the following control variables were included in our regression model: age, marital status (0 = married, living separated from spouse, divorced, widowed, single, 1 = married; living together with spouse), self-rated health (ranging from 1= “very good” to 5 = “bad”), and the presence of severe disability (yes/no). In sensitivity analysis, log equivalent net income was added to the main model. 

### 2.4. Statistical Analysis

To estimate the association between personality factors and sick leave days, Poisson fixed effects (FE) regressions were used. Poisson FE regressions were used because they provide consistent estimates even when unobserved factors (e.g., genetic disposition) are associated with the explanatory variables (under the assumption of strict exogeneity). Consequently, time-constant factors (both, observed and unobserved) associated with the explanatory variables do not bias the FE estimates [15].

FE regressions solely exploit variations within units (i.e., individuals) over time. In our current study, we investigated whether changes within individuals over time in personality factors were associated with changes within individuals over time in the number of sick leave days. 

Therefore, only time-varying factors (such as self-rated health) can be used as main effects in FE regressions. This means that time-constant factors (i.e., factors that do not vary within individuals over time such as sex, ethnicity or genetic factors) cannot be included as main effects in Poisson FE regressions. However, these time-constant factors are implicitly controlled [15]. The choice of using FE regressions was also corroborated by the Hausman test (Hausman test statistic= 958.83, *p* < 0.001). The level of significance was set at 0.05. Statistical analyses were carried out with Stata 15.0 (Stata Corp., College Station, TX, USA).

### 2.5. Ethical Approval and Consent to Participate

Not applicable. An ethical approval was not obtained because criteria for the need of an ethical statement were not met (risk for the respondents, lack of information about the aims of the study, examination of patients). However, the German Council of Science and Humanities (Wissenschaftsrat) evaluated the German Socio-Economic Panel (GSOEP) at the Deutsches Institut für Wirtschaftsforschung, (DIW), Berlin. The German Council of Science and Humanities approved the GSOEP.

## 3. Results

### 3.1. Sample Characteristics

Pooled over time, descriptive characteristics of the individuals included in FE regressions are shown in Table 1. The final study sample consisted of 16,344 observations (6594 individuals), mean age was 42.91 (SD: 10.81), the majority were married (62.21%). The average scores for personality factors in the full sample were as follows: neuroticism—11.43 (SD: 3.58), extraversion—14.55 (SD: 3.40), openness to experience—13.50 (SD: 3.45), agreeableness—15.95 (SD: 2.88), and conscientiousness—17.65 (SD: 2.63). The average number of sick leave days in the preceding year was 11.12 (SD: 26.81). 

### 3.2. Regression Analysis

Prior to FE regression analysis, it was checked whether there is enough within-variation (i.e., changes within individuals) in the independent variables and the outcome measure over time (from 2005 to 2013) to get precise estimates. To this end, the Stata commands ‘xttab’ and ‘xttrans’ were used. Actually, there were enough within-variation in all variables (results not shown, but available upon request). Therefore, FE regressions were performed with the aforementioned time-varying variables.

The results of the FE regression are presented in Table 2. By using FE regressions, we examined whether intraindividual changes in personality characteristics were associated with intraindividual changes in the number of sick leave days. 

In the main model, the FE regression showed that the number of sick leave days within individuals over time increased with increasing neuroticism within individuals over time (from 2005 to 2013) (incidence rate ratio: 1.02 [95%-CI: 1.00–1.04], *p* < 0.05). The other personality factors were not significantly associated with the number of sick leave days. 

The robustness of our relation of interest was examined by comparing the main model with an additional model (in terms of significance and effect size; results not shown, but available upon request). In this additional model, income was added to the main model. FE regressions showed that the effect of neuroticism on sick leave days remained virtually the same (β = 1.02, [1.00–1.04], *p* < 0.05). 

## 4. Discussion

### 4.1. Main Findings

Based on a nationally representative longitudinal sample (GSOEP), the aim of the current study was to investigate whether changes in personality are associated with changes in sick leave days. Adjusting for potential confounders, Poisson FE regressions showed that increases in neuroticism were associated with increased sick leave days. Other personality factors were not significantly associated with sick leave days.

### 4.2. Comparison with the literature and Possible Explanations

Comparing the results of the current study with the previous research is difficult because of the lack of studies in this research area investigating personality factors and sick leave days and additionally, as mentioned earlier, because most studies in this area are cross-sectional; comparing the results from single points in time, obtained from cross-sectional studies, with results from longitudinal studies, representing personal or social changes, is difficult. Most of the cross-sectional studies found that personality factors, for the most part, were not associated with the number of sick leave days [8,9,10]; the only factor that was strongly associated with sick leave days was neuroticism [9,10]. For example, Störmer and Fahr [9] found, in their cross-sectional study, a significant association between neuroticism and absenteeism (sickness absence with more than 30 days was excluded). They observed that neurotic male employees have 0.47 additional days of absence. However, this association was not observed among women.

Furnham and Miller [8] found that none of the personality factors, which was measured with Eysenck Personality Inventory (extraversion, neuroticism) were associated with actual recorded sick leave days, only with the number of occasions on which each person had been absent. The dependent variable “Absenteeism” was defined as periods of absence during the year and actual recorded sick days per annum. The results of this study are difficult to compare with the results of the current work, because of the study design (cross-sectional vs. longitudinal) and the tools used for measuring personality are different. Vlasveld et al. [10] investigated the association between the “Big Five” factors and sickness absence in two groups of individuals: healthy subjects and subjects with psychopathology (depressive and anxiety disorders). According to the results, a higher value of neuroticism was associated with short-term absence (for the healthy group) and with long-term absence (for both groups). 

The only study investigating the impact of personality on sick leave days longitudinally was the study of Kok et al. [11]; using multiple logistic regression they examined conscientiousness and revealed that it does not affect the long-term absenteeism of employees (Netherlands Study of Depression and Anxiety). The current study used a Poisson FE regression that unlike multiple logistic regression allows to investigate the relation between intraindividual changes in personality and it allows to mitigate the problem of unobserved heterogeneity. Our findings add to this longitudinal study by examining the personality factors in a broader sense. In other words: This is the first study investigating the relation between intraindividual changes in personality (measured with the Big Five) and changes in sick leave days.

In this study, increased neuroticism led to an increase in the number of sick leave days. One explanation approach might be that individuals who score high in neuroticism are very concerned about his or her ailments and consequently talk with the peer group about these ailments. The peer group might urge the individuals scoring high in neuroticism to visit the doctor who finally give him or her a sick note. However, future research is required to clarify these assumptions. Another explanation might be that neuroticism is directly associated with mental health [16,17], particularly with mental illnesses such as depression and anxiety [18,19], which in turn leads to (often longer [20,21]) absence on sick leave [22,23,24]. 

### 4.3. Strengths and Limitations

This is one of the first studies investigating the relation between personality factors and sick leave days longitudinally. Data were derived from a population-based, longitudinal study of German households (GSOEP). Using Poisson FE regression, intraindividual changes were examined and the problem of unobserved heterogeneity was reduced [15]. There is the possibility of endogeneity (reverse causality) [25]. Thus, it is, for example, possible that sick leave days experienced ten months ago increased the present level of neuroticism. Consequently, further research is required based on panel instrumental variable (IV) approaches in order to deal with endogeneity problems between personality factors and sick leave days. However, the consistency of IV estimates depends on accurate instruments. IV estimates relying on weak instruments lead to highly biased estimates. Consequently, Poisson FE regressions were used in this study. The BFI-S was used to quantify personality. Generally, it has satisfactory psychometric properties. However, it is worth noting that it has shortcomings in agreeableness [14]. Furthermore, we cannot dismiss the possibility that there is some recall bias regarding the number of sick leave days in the preceding year. 

## 5. Conclusions

The findings of the current study highlight the association between neuroticism and sick leave days longitudinally. Further research is required to elucidate the underlying mechanisms. 

## Figures and Tables

**Table 1:** Sample characteristics for individuals included in conditional fixed effect logistic regressions (*n* = 16,344; year 2005, 2009, 2013).

Variables	N/Mean	%/(SD)
Age in years	42.91	(10.81)
Marital status:		
- Married and living separated from spouse/divorced/widowed/single	6177	37.79%
- Married and living together with spouse	10,167	62.21%
Presence of severe disability:		
- No	15,202	93.01%
- Yes	1142	6.99%
Self-rated health		
(1 = “very good” to 5 = “bad”)	2.51	(0.84)
Big Five:		
- Neuroticism (higher values signify higher neuroticism)	11.43	(3.58)
- Extraversion (higher values signify higher extraversion)	14.55	(3.40)
- Openness to experience (higher values signify higher openness to experience)	13.50	(3.45)
- Agreeableness (higher values signify higher agreeableness)	15.95	(2.88)
- Conscientiousness (higher values signify higher conscientiousness)	17.65	(2.63)
Number of sick leave days in the preceding year	11.12	26.81

**Table 2:** Results of Poisson FE regression models with number of sick leave days in the preceding year as outcome measure; year 2005, 2009, and 2013.

Independent Variables	Outcome Measure
	Number of Sick Leave Days
Neuroticism (higher values signify higher neuroticism)	1.02 * (1.00–1.04)
Extraversion (higher values signify higher extraversion)	1.02 ^+^ (1.00–1.04)
Openness to experience (higher values signify higher openness to experience)	0.99 (0.97–1.01)
Agreeableness (higher values signify higher agreeableness)	1.00 (0.98–1.02)
Conscientiousness (higher values signify higher conscientiousness)	0.99 (0.96–1.01)
Age	1.04 *** (1.03–1.05)
Marital status: married, living together with spouse (Ref.: married and living separated from spouse, divorced, widowed, single)	1.04 (0.90–1.22)
Presence of severe disability (Ref.: no)	1.86 *** (1.49–2.27)
Self-rated health (from 1 = “very good” to 5 = “bad”)	1.54 *** (1.43–1.65)
Observations	16,344
Number of individuals	6594

Comments: Incidence rate ratios were reported; cluster-robust 95% confidence intervals in parentheses; *** *p* < 0.001, ** *p* < 0.01, * *p* < 0.05, ^+^ *p* < 0.10

## References

[1] Duckworth A.L., Weir D.R., Tsukayama E., Kwok D. (2012). Who does well in life? Conscientious adults excel in both objective and subjective success. Front. Psychol..

[2] Nyhus E.K., Pons E. (2005). The effects of personality on earnings. J. Econ. Psychol..

[3] Sutin A.R., Costa P.T., Miech R., Eaton W.W. (2009). Personality and career success: Concurrent and longitudinal relations. Eur. J. Personal..

[4] Burke R.J., Matthiesen S.B., Pallesen S. (2006). Personality correlates of workaholism. Personal. Individ. Differ..

[5] Bakker A.B., Van Der Zee K.I., Lewig K.A., Dollard M.F. (2006). The relationship between the big five personality factors and burnout: A study among volunteer counselors. J. Soc. Psychol..

[6] Judge T.A., Higgins C.A., Thoresen C.J., Barrick M.R. (1999). The big five personality traits, general mental ability, and career success across the life span. Pers. Psychol..

[7] Boyce C.J., Wood A.M., Powdthavee N. (2013). Is personality fixed? Personality changes as much as “variable” economic factors and more strongly predicts changes to life satisfaction. Soc. Indic. Res..

[8] Furnham A., Miller T. (1997). Personality, absenteeism and productivity. Personal. Individ. Differ..

[9] Störmer S., Fahr R. (2013). Individual determinants of work attendance: Evidence on the role of personality. Appl. Econ..

[10] Vlasveld M.C., van der Feltz-Cornelis C.M., Anema J.R., van Mechelen W., Beekman A.T., van Marwijk H.W., Penninx B.W. (2013). The associations between personality characteristics and absenteeism: A cross-sectional study in workers with and without depressive and anxiety disorders. J. Occup. Rehabil..

[11] Kok A.A., Plaisier I., Smit J.H., Penninx B.W. (2017). The impact of conscientiousness, mastery, and work circumstances on subsequent absenteeism in employees with and without affective disorders. BMC Psychol..

[12] Wagner G.G., Frick J.R., Schupp J. (2007). The German Socio-Economic Panel Study (Soep)-Evolution, Scope and Enhancements.

[13] Rammstedt B., Koch K., Borg I., Reitz T. (2004). Entwicklung und validierung einer kurzskala für die messung der big-five-persönlichkeitsdimensionen in umfragen. Zuma Nachr..

[14] Dehne M., Schupp J. (2007). Persönlichkeitsmerkmale im sozio-oekonomischen panel (soep)-konzept, umsetzung und empirische eigenschaften. Res. Notes.

[15] Brüderl J. (2010). Kausalanalyse mit paneldaten. Handbuch der Sozialwissenschaftlichen Datenanalyse.

[16] Furnham A., Cheng H. (1999). Personality as predictor of mental health and happiness in the east and west. Personal. Individ. Differ..

[17] Lu L. (1994). University transition: Major and minor life stressors, personality characteristics and mental health. Psychol. Med..

[18] Farmer A., Redman K., Harris T., Mahmood A., Sadler S., Pickering A., McGUFFIN P. (2002). Neuroticism, extraversion, life events and depression. Br. J. Psychiatry.

[19] Jylhä P., Isometsä E. (2006). The relationship of neuroticism and extraversion to symptoms of anxiety and depression in the general population. Depress. Anxiety.

[20] Knudsen A.K., Harvey S.B., Mykletun A., Øverland S. (2013). Common mental disorders and long-term sickness absence in a general working population. The hordaland health study. Acta Psychiatr. Scand..

[21] Savikko A., Alexanderson K., Hensing G. (2001). Do mental health problems increase sickness absence due to other diseases?. Soc. Psychiatry Psychiatr. Epidemiol..

[22] Duijts S.F., Kant I., Swaen G.M., van den Brandt P.A., Zeegers M.P. (2007). A meta-analysis of observational studies identifies predictors of sickness absence. J. Clin. Epidemiol..

[23] Laitinen-Krispijn S., Bijl R. (2000). Mental disorders and employee sickness absence: The nemesis study. Soc. Psychiatry Psychiatr. Epidemiol..

[24] Nieuwenhuijsen K., Verbeek J.H., de Boer A.G., Blonk R.W., van Dijk F.J. (2006). Predicting the duration of sickness absence for patients with common mental disorders in occupational health care. Scand. J. WorkEnviron. Health.

[25] Roberts M.R., Whited T.M. (2012). Endogeneity in empirical corporate finance. Handbook of the Economics of Finance (Volume 2A).

